# Application of 2-step percutaneous transhepatic choledochoscopic lithotomy in elderly patients diagnosed with complex intrahepatic or extrahepatic cholelithiasis: A case series study

**DOI:** 10.1097/MD.0000000000047927

**Published:** 2026-02-28

**Authors:** Pan Liu, Ji-Lin Zhang, Sheng Yu, Shun-Hai Liu, Xin Xiang

**Affiliations:** a Department of Hepatobiliary Surgery, The First People’s Hospital of Neijiang, Neijiang, Sichuan, China; b Department of Hepatobiliary Surgery, The People’s Hospital of Neijiang Dongxing District, Neijiang, Sichuan, China.

**Keywords:** extrahepatic cholelithiasis, intrahepatic cholelithiasis, percutaneous transhepatic choledochoscopic lithotomy

## Abstract

**Rationale:**

The optimal management of complex intrahepatic and extrahepatic cholelithiasis in very elderly patients with prior abdominal surgery remains challenging. This case series aims to report the application and feasibility of a 2-step percutaneous transhepatic choledochoscopic lithotomy (PTCSL) approach in this high-risk population.

**Patient concerns:**

Fourteen patients aged 75 years or older with complex cholelithiasis (widespread or large calculi) and a history of upper abdominal surgery were included.

**Diagnoses:**

All patients were diagnosed with complex intrahepatic or extrahepatic cholelithiasis based on imaging findings.

**Interventions:**

All patients underwent a planned 2-step procedure. The first step involved percutaneous transhepatic cholangial drainage (PTCD) to achieve biliary decompression. The second step consisted of definitive PTCSL for stone removal.

**Outcomes:**

Four (29%) patients admitted for Reynolds’ pentad underwent bedside PTCD under ultrasound guidance in an emergency setting. Nine (64%) with a combination of ultrasonography and cholangiography. The interval from the first-step PTCD to the second-step PTCSL ranged from 15 to 96 days (mean, 53.6 days) for all patients. Twelve (86%) patients underwent 1 or 2 surgeries for complete stone removal. All complications were manageable.

**Lessons:**

This small-scale case series suggests that the 2-step PTCSL approach is a safe and feasible therapeutic alternative for selected elderly and high-risk patients with complex cholelithiasis who are poor candidates for conventional surgery. These preliminary findings warrant further validation in larger prospective studies.

## 1. Introduction

Intrahepatic or extrahepatic cholelithiasis is a common biliary disease in East Asian countries. The etiology of this disease is associated with biliary tract infections, biliary ascariasis, biliary duct malformations and stenosis. Furthermore, its morbidity may be correlated with lower socioeconomic status and rural settings.^[1,2]^ Previous research has indicated that approximately 20% to 45% of patients undergoing biliary surgery have hepatolithiasis.^[3,4]^ It is the leading cause of mortality among patients with nontumor biliary diseases and results in a diminished quality of life, particularly among those experiencing recurrent bacterial cholangitis and undergoing multiple surgeries.^[1,2]^

Treatment for intrahepatic and extrahepatic cholelithiasis primarily involves surgical intervention, which includes endoscopic retrograde cholangiopancreatography (ERCP), choledocholithotomy, and hepatectomy.^[1,5]^ However, in clinical management, a substantial number of elderly patients exhibit reluctance toward open abdominal procedures, particularly those with a history of multiple prior surgeries. Additionally, ERCP proves clinically inapplicable for patients presenting with multiple intrahepatic bile duct stones or those who have undergone choledochojejunostomy. These clinical challenges have resulted in repeated conservative treatment approaches for geriatric patients with complex hepatobiliary calculi during disease exacerbations. This management pattern not only elevates hospitalization frequency but also potentially leads to fatal outcomes when conservative strategies ultimately fail to control disease progression.

Percutaneous transhepatic choledochoscopic lithotomy (PTCSL), a minimally invasive “upward approach” for stone removal, has been increasingly adopted in recent years.^[6–8]^ PTCSL avoids open surgery.^[9]^ It encompasses 1-step and 2-step approaches. Although the safety of 1-step PTCSL has been established in the general population,^[4,8,10]^ there is a scarcity of studies directly comparing 1-step and 2-step PTCSL, particularly in elderly patients. Since initiating PTCSL in 2020, our institution has predominantly adopted the 2-step protocol for geriatric patients. This strategic shift is based on safety considerations. This strategic shift addresses the frequent presentation of complex calculus distribution and severe cholangitis manifestations in this population at admission. Herein, we present a retrospective analysis of clinical and follow-up data from 14 elderly (≥75 years) patients with complicated intrahepatic or extrahepatic cholelithiasis successfully managed via 2-step PTCSL at our center.

## 2. Patients and methods

### 2.1. Patients

This study was approved by the Ethics Committee of the First People’s Hospital of Neijiang (Sichuan, China) (Approval No. 2024-33). Given the retrospective nature of this study, the Ethics Committee waived the requirement for informed consent. All methods were carried out in accordance with relevant guidelines and regulations. We performed a retrospective review of medical records for patients diagnosed at our hospital between January 2020 and August 2024. The final study sample included those who fulfilled predefined inclusion and exclusion criteria. The inclusion criteria were as follows: age ≥75 years; diagnosed with intrahepatic or extrahepatic cholelithiasis through imaging examinations, including abdominal ultrasound, abdominal computed tomography, or abdominal magnetic resonance cholangiopancreatography; underwent a 2-step PTCSL; history of ≥1 upper abdominal surgeries; and a distal intrahepatic bile duct diameter ≥3 mm. The exclusion criteria were as follows: obvious liver lobe atrophy; imaging indicating ≥3 intrahepatic bile duct strictures; underwent alternative stone removal methods after the 1st-step percutaneous transhepatic cholangial drainage (PTCD), such as ERCP, choledocholithotomy, or hepatectomy; liver function remained grade C despite active treatment; and general anesthesia contraindicated due to high risk.

Ultimately, the final study sample comprised 14 patients (5 male, 9 female; mean [± standard deviation] age, 80.8 years ± 4.1 years; median age, 81.5 years [range, 75–86 years]) (Table 1).

**Table 1:** The basic information, clinical symptoms, and relevant results of the 1st-step PTCD for the patients.

Patient no.	Sex	Age (yr)	Clinical symptoms	Child-Pugh grade	Procalcitonin (ng/ml)	Quantity and distribution of calculi	Complications of other organs	Puncture timing	Guidance method	Puncture position
1	F	80	Charcot triad	B	37.8	Multiple; CBD	Liver abscess; AHF	Selective	UG-PTC	B5
2	M	75	Reynolds pentad	C	56.1	Multiple; CBD	AKI	Emergency	UG	B3
3	M	82	Charcot triad	B	12.4	Multiple; HBD/CHD/CBD	AP; hydrothorax; ascites; pulmonary infection	Selective	UG-PTC	B3
4	F	81	Charcot triad	A	6.2	Complete filling; RPSD/CBD	Liver abscess	Selective	UG-PTC	B3
5	M	86	Reynolds pentad	B	7.9	Multiple; CBD	AP; AKI; nutritional anemia	Emergency	UG	B5
6	F	75	Charcot triad	A	1.8	Multiple; RPSD/CBD	No	Selective	UG-PTC	B3
7	F	82	Abdominal pain	A	13.3	Complete filling; LHD	Liver abscess	Selective	UG-PTC	B3
8	M	80	Charcot triad	C	16.3	Multiple; CBD	AKI; peritonitis; intestinal obstruction	Selective	UG-PTC	B3
9	F	83	Reynolds pentad	B	4.6	Multiple; LHD/CBD	AKI	Emergency	UG	B3
10	F	76	Charcot triad	B	16.3	Diffuse; hepatolithiasis	No	Selective	UG-PTC	B8
11	M	75	Reynolds pentad	B	>100	Multiple; HBD	AKI	Emergency	UG	B3
12	F	85	Charcot triad	A	7.4	Complete filling; hepatolithiasis/CHD/CBD	No	Selective	UG	B8
13	F	85	Charcot triad	B	3.1	Complete filling; HBD/CHD/CBD	AP	Selective	UG-PTC	B3
14	F	86	Charcot triad	B	3.5	Multiple; CBD	AP; AHF	Selective	UG-PTC	B3

AHF = acute heart failure, AKI = acute kidney injury, AP = acute pancreatitis, CBD = common bile duct, CHD: common hepatic duct, HBD: hilar bile duct, LHD = left hepatic duct, PTCD = percutaneous transhepatic cholangial drainage, RPSD = right posterior segmental hepatic ducts, UG = ultrasound-guided, UG-PTC = ultrasound-guided percutaneous transhepatic cholangiography.

### 2.2. Procedure details

#### 2.2.1. First-step: PTCD

**Figure 1.**
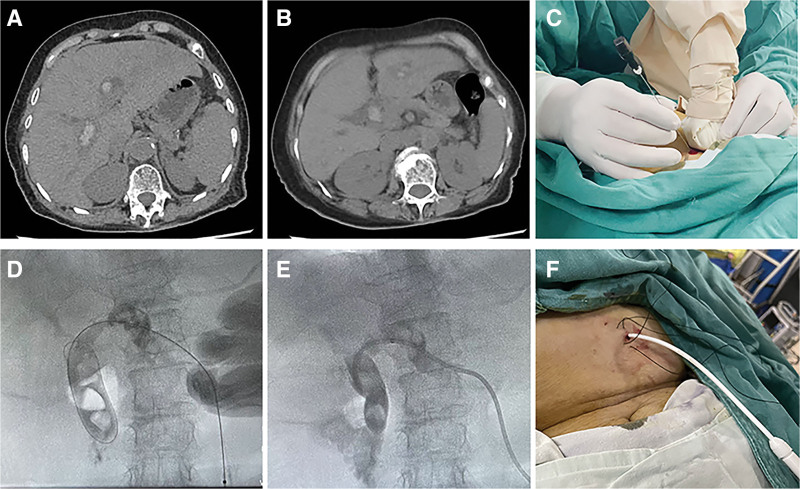
Preoperative abdominal CT scan confirming biliary stone distribution and anticipated puncture pathway in a 78-year-old female patient (A and B). Successful intraoperative placement of an 8.5 Fr drainage catheter into the common bile duct under combined ultrasound and cholangiography guidance (C–F). CT = computed tomography.

**Figure 2.**
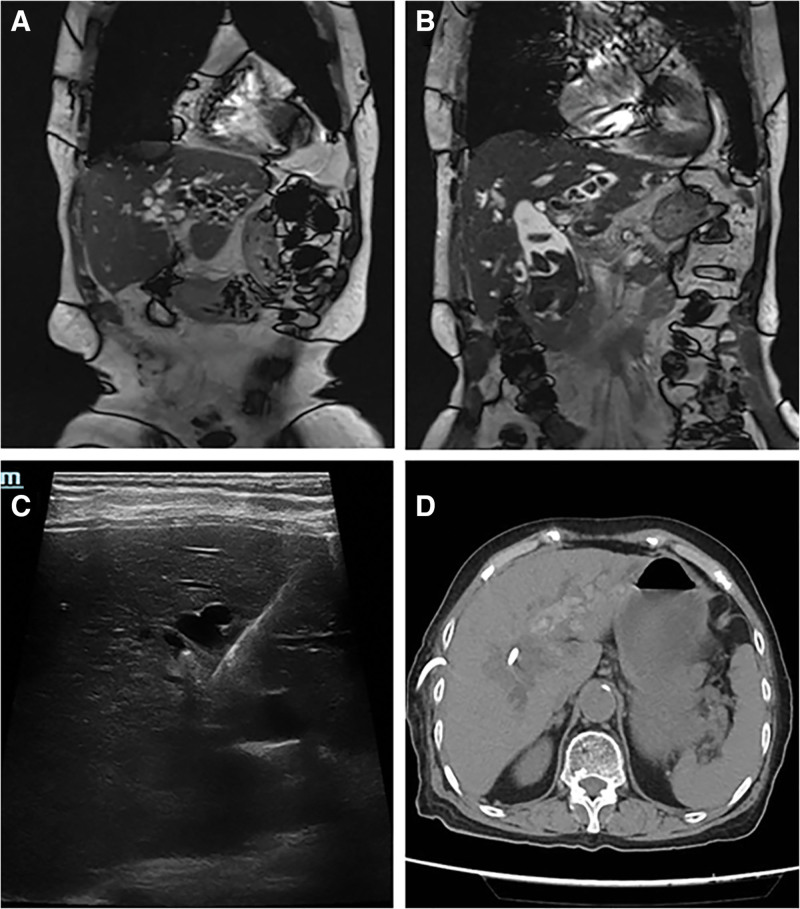
Diagnostic imaging demonstrating biliary calculus characteristics in an 86-year-old female patient (A and B). Emergency bedside ultrasound-guided drainage catheter placement (C), followed by postoperative abdominal CT verification of catheter positioning (D). CT = computed tomography.

There are 2 guiding methods for the 1st-step of PTCD: ultrasound combined with cholangiography and ultrasound guidance alone. If the patient did not require emergency treatment and was assessed to be suitable for subsequent PTCSL, the 1st-step PTCD was guided by ultrasound combined with cholangiography by reviewing imaging data to clarify the distribution of stones and intrahepatic bile duct dilatation, preselecting the puncture point and angle that could be used for the 2nd-step PTCSL, puncturing the intrahepatic bile duct under ultrasound guidance, and placing the drainage tube (8.5 Fr, Cook Medical, Bloomington) into the common bile duct or across the bilioenteric anastomosis (if present) under the guidance of cholangiography (Fig. 1). After firmly securing the drainage tube, the procedure was concluded, and continuous drainage of the PTCD tube was maintained until the patient cholangitis improved before attempting to clamp the drainage tube. If the patient required emergency treatment and was deemed unsuitable for ERCP, an ultrasound-guided PTCD procedure was performed at the bedside. Under ultrasound guidance, a safe and simple path was selected, and continuous biliary drainage was maintained after placement of the drainage tube (6/8 Fr, Zhengzhou DIALL Medical Technology Co., Ltd, Zhengzhou, Henan, China) (Fig. 2). All patients were discharged with a PTCD tube after their physical condition improved.

#### 2.2.2. Second-step: PTCSL

**Figure 3.**
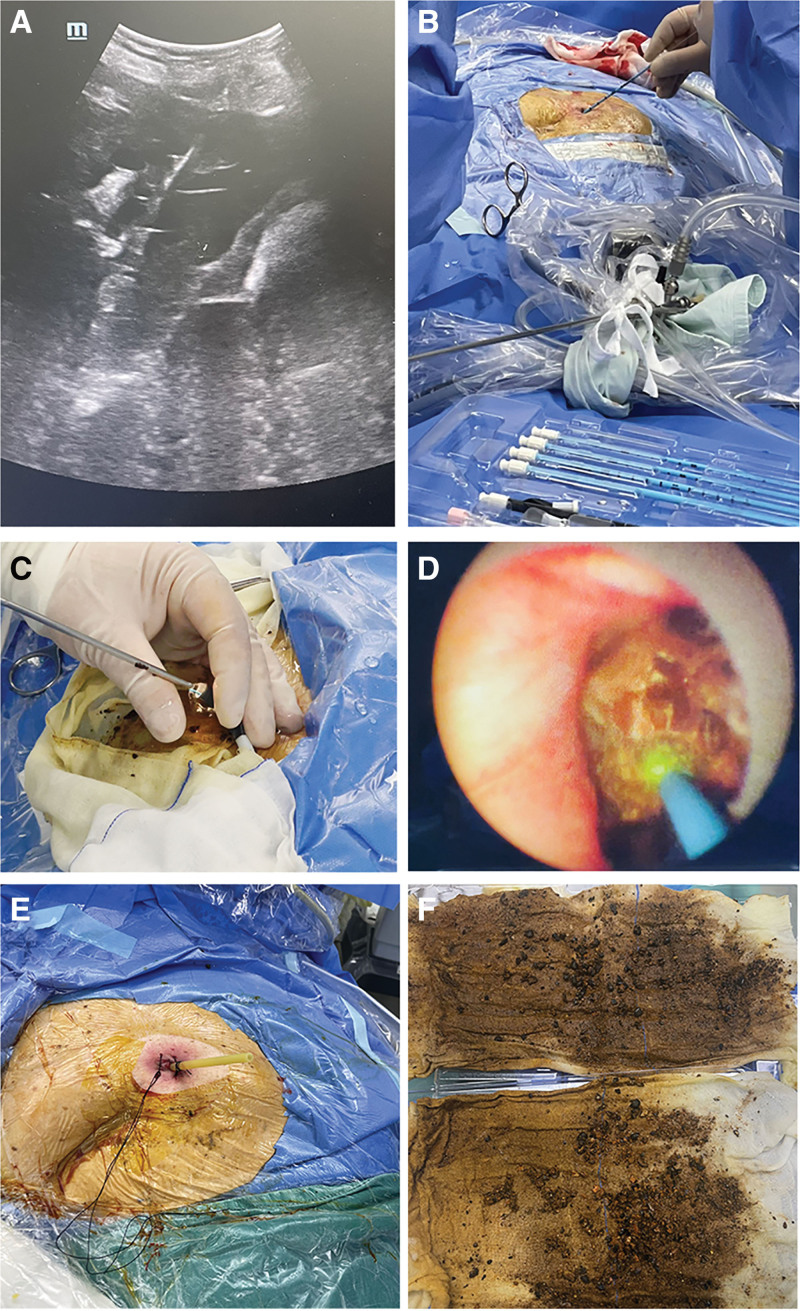
Procedural workflow: (A) ultrasound-guided guidewire insertion, (B) sequential tract dilation using dilatation kit, (C and D) holmium laser lithotripsy under rigid choledochoscopic guidance, and (E and F) final drainage catheter placement and calculus collection through established tract.

After a 2-week recovery period following the 1st-step PTCD, patients underwent a comprehensive evaluation to determine their suitability for the 2nd-step PTCSL. All patients underwent PTCSL under general anesthesia, and the entire procedure was strictly performed within 3 hours. Guided by intraoperative ultrasound, a guidewire was inserted through the PTCD tube into the intrahepatic bile duct, followed by the removal of the PTCD tube. A minimally invasive dilation set (Percutaneous Nephrostomy Set, 4KQDSZ, Zhejiang Huamei Medical Equipment Co., Ltd., Ningbo, Zhejiang, China) was then used to gradually expand the sinus tract along the guidewire until it reached 18 Fr, at which point a protective sheath was placed. Subsequently, a rigid nephroscope (89.101; Richard Wolf GmbH, Knittlingen, Baden-Wuerttemberg, Germany) was used to reach the target bile duct, and saline solution was continuously infused into the intrahepatic bile duct using an adjustable pressure pump (type APL; Guangzhou Jielun Medical Equipment Co., Ltd., Guangzhou, Guangdong, China). Once the stones were located, a rigid ureteroscope coupled with holmium laser lithotripsy (ACU-H2, Accu-Tech Co., Ltd., Beijing, China) and saline irrigation were used for stone fragmentation and removal. If necessary, a net basket (MD-A-RN-N201818, Zhejiang Chuangxiang Medical Technology Co., Ltd., Hangzhou City, Zhejiang, China) and a clamp (5 Fr, Richard Wolf GmbH, Knittlingen, Germany) were used to crush or extract the stones. In cases for which the stones were inaccessible to a rigid nephroscope, such as in the common bile duct, an electronic choledochoscope (CHF-V; Olympus, Tokyo, Japan) was used to complete the procedure. If biliary tract stenosis was found during the procedure, a rigid nephroscope was used in conjunction with a guidewire to complete blunt dilatation. Ultrasonography was used during the procedure to determine whether all stones had been removed. After stone removal was completed, an 18/16 Fr drainage tube was placed along the sinus tract into the common bile duct (if possible, the drainage tube passed through the stenosis of the intrahepatic bile duct) or through a biliary-enteric anastomosis (if present). Subsequently, the drainage tube was fixed and connected to a drainage bag to complete the surgery (Fig. 3).

#### 2.2.3. Subsequent treatment

All patients underwent a follow-up upper abdominal computed tomography scan and laboratory investigations on postoperative day 1 to clarify postoperative status. If the stones were not completely removed, further surgery was considered 1 week later. If the stones were completely removed, the biliary drainage tube was clipped approximately 3 days postsurgery and was removed approximately 1-week postsurgery once the drainage tube could be completely clipped. If significant intrahepatic bile duct stenosis or biliary-enteric anastomotic stenosis was evident, it was recommended that the patient wear the drainage tube for 4 to 12 weeks, depending on the severity of the stenosis.

After discharge, patients were advised to take ursodeoxycholic acid^[11,12]^ as a long-term treatment and to undergo long-term follow-up at a hepatobiliary surgery clinic. The routine follow-up interval was every 3 months, which included liver function tests and abdominal ultrasound examinations.

### 2.3. Statistical analysis

Data extraction from electronic medical records was performed independently by 2 authors (Pan Liu and Ji-Lin Zhang) to ensure accuracy. Any discrepancies were resolved through discussion with a senior author (Xin Xiang). All statistical analyses were descriptive in nature and were prespecified prior to data collection to summarize patient characteristics and outcomes. Qualitative data are expressed as raw count, proportion, and percentage. Quantitative data are reported as mean and/or median, standard deviation, interquartile range, and range.

## 3. Results

### 3.1. Study population and the 1st-step PTCD outcomes

Among the 14 patients, the majority (13/14, 93%) presented with signs of systemic infection, including 9 (64%) with Charcot triad and 4 (29%) with the more severe Reynolds pentad. This high prevalence of severe cholangitis upon admission underscores the critical condition of this cohort and the urgent need for effective biliary decompression, which was achieved via the 1st-step PTCD. The 4 patients with Reynolds pentad underwent emergency ultrasound-guided PTCD. Of the remaining 10 patients, 9 (64%) underwent PTCD guided by ultrasound combined with cholangiography, while the other patient (7%) underwent selective ultrasound-guided PTCD only due to an excessive number of hepatolithiasis and choledocholithiasis. All patients (14/14, 100%) underwent ≥ 1 biliary surgery. Five patients (36%) were diagnosed with simple choledocholithiasis, 8 (57%) had both hepatolithiasis and choledocholithiasis, and 1 (7%) had only hepatolithiasis. Three (21%) patients had no significant comorbidities, whereas 11 (79%) had significant comorbidities. Ten patients (71%) had a puncture point in segment 3, 2 (14%) in segment 5, and 2 (14%) in segment 8. Depending on the severity of their condition, patients were discharged from the hospital 1 to 13 days (mean length of stay, 5.4 ± 3.6 days) after the 1st-step PTCD surgery. The baseline characteristics and relevant results of the 1st-step PTCD for the patients are summarized in Table 1.

### 3.2. Interval from 1st-step PTCD to 2nd-step PTCSL

The interval from the 1st-step PTCD to the 2nd-step PTCSL ranged from 15 to 96 days (mean, 53.6 days) for all patients. Among these, some patients, especially patients 12 and 14, chose to keep the PTCD tube for a longer period rather than undergo PTCSL as soon as possible due to psychological factors, such as no recurrence of cholangitis after the placement of the PTCD tube and fear of general anesthesia surgery, rather than failing the physical assessment. Three (21%) patients experienced complications related to the PTCD tube while carrying the tube outside the hospital, including 1 case of tube obstruction, 1 case of tube dislodgement, and 1 case of persistent pain at the puncture site of the drainage tube. Notably, the tube obstruction occurred in a patient who had undergone emergency PTCD with a 6 Fr catheter, suggesting that a smaller tube size may be a potential risk factor. Patients with drainage tube obstruction and dislodgement underwent drainage tube replacement along the previous sinus tract, and patients with pain underwent a 2nd-step PTCSL 15 days after the placement of the PTCD tube. Information regarding the period from the 1st-step PTCD to the 2nd admission is shown in Table 2.

**Table 2:** The relevant outcomes for patients from the 1st-step PTCD to the completion of the 2nd-step PTCSL.

Patient no.	Duration between PTCD and 1st discharge; (d)	Duration between PTCD and PTCSL; (d)	Complications related to PTCD tube	Complications related to PTCSL	Intra-operative blood loss (mL)	Number of PTCSL surgery	Completely remove stones in 1st operation?	Completely removed stones in the end?	Operative time (1st PTCSL surgery) (min)	Biliary stenosis present?
1	3	42	No	No	5	1	Yes	Yes	142	No
2	13	32	No	No	5	1	Yes	Yes	160	No
3	7	47	No	No	5	1	Yes	Yes	175	No
4	2	68	No	Postoperative watery diarrhea	5	1	No	No	180	No
5	5	51	Tube obstruction	No	5	1	Yes	Yes	170	No
6	3	37	No	No	5	1	Yes	Yes	115	No
7	4	35	No	No	5	1	Yes	Yes	160	Yes
8	6	75	No	Sinus hemorrhage	50	2	No	Yes	180	No
9	12	74	No	No	5	2	No	Yes	180	No
10	7	15	Local skin pain	No	5	1	Yes	Yes	146	Yes
11	7	54	No	No	5	1	No	Yes	180	Yes
12	2	86	No	No	5	2	No	No	172	Yes
13	1	39	Tube dislodgement	Intraoperative hypothermia	5	2	No	Yes	148	No
14	3	96	No	No	5	2	No	Yes	175	No

PTCD = percutaneous transhepatic cholangial drainage, PTCSL = percutaneous transhepatic choledochoscopic lithotomy.

### 3.3. Outcome of the 2nd-step PTCSL and clinical effect

The procedural details and patient outcomes are comprehensively detailed in Table 2. Three (21%) patients experienced complications related to PTCSL; more specifically, postoperative watery diarrhea, bleeding during sinus tract dilation, and intraoperative hypothermia. The ultimate stone clearance rate was 86% (12/14), achieved after 1 or 2 PTCSL sessions. Seven patients (50%) achieved complete clearance in a single procedure, while 4 (29%) required a 2nd session. Notably, in patient 11, stones that were not completely removed during the 1st lithotomy were completely expelled by 30-day follow-up with the aid of an 18 Fr drainage tube and oral ursodeoxycholic acid. The 2 cases of incomplete clearance were due to patient-specific factors rather than technical failure: Patient 4 declined further hospitalization, while patient 13, with excessive stones and poor baseline status, opted to cease treatment after the right-sided and common bile duct stones were cleared, leaving manageable left-sided stones.

### 3.4. Long-term follow-up

According to discharge instructions, 8 patients (57%) were actively followed-up with valid data. Among these, 2 (25%) experienced stone recurrence at 3 and 9 months postoperatively, respectively. Two patients died of other diseases at 14 and 19 months postoperatively, without experiencing stone recurrence before their deaths. The remaining 6 patients were followed-up via telephone. Two patients died approximately 1-year postoperatively, although the cause remains unknown. The remaining 4 patients reported no significant abdominal pain or jaundice but did not undergo imaging examination.

## 4. Discussion

Intrahepatic or extrahepatic cholelithiasis is a complex disorder with a notably high incidence in China.^[2]^ Based on the present investigation, involving 14 elderly patients, 2-step PTCSL was a feasible therapeutic alternative for elderly individuals experiencing exacerbations of acute cholangitis and a history of multiple surgeries.

In elderly patients experiencing recurrent stone formation, the prolonged presence of stones and multiple surgical histories often lead to significant damage to their biliary tract structures, thereby complicating the distribution of stones within the biliary tract.^[13]^ Consequently, ERCP for stone removal can be challenging for some of these patients. Additionally, in elderly patients with a history of biliary surgery, abdominal adhesions and their physical condition at the time of onset pose certain difficulties in promptly performing biliary tract incision and stone removal under general anesthesia. Furthermore, elderly patients tend to exhibit rapid disease progression upon onset, which can readily result in multiorgan dysfunction. Failure to perform biliary tract decompression promptly may pose a risk for mortality in these patients.^[9]^

PTCD, a surgical procedure performed under local anesthesia, rapidly achieves biliary decompression.^[14]^ In addition, when biliary tract radiography was performed, the distal end of the drainage tube was positioned accurately within the common bile duct. Once the PTCD tube is in place, it can be safely clamped without increasing the risk for cholangitis, thereby mitigating fluid bile leakage and appetite loss, and facilitating better conditions for subsequent surgery. Furthermore, biliary tract radiography offers intuitive visualization of biliary structures,^[7]^ while a mature sinus tract facilitates subsequent procedures. Given these advantages, despite evidence supporting the effectiveness, safety, and cost-efficiency of 1-step PTCSL,^[4,8,10]^ our center prioritizes the 2-step approach for elderly patients, primarily for safety reasons. However, there is a dearth of comparative studies investigating 1- versus 2-step PTCSL in elderly patients.

Among the 14 patients enrolled in the present study, the initial placement of the PTCD tube was successfully performed without any notable complications because all patients exhibited significantly dilated intrahepatic bile ducts, consistent with findings reported by Pedersoli et al.^[15]^ Nevertheless, during the interval between the completion of PTCD tube placement and the subsequent PTCSL procedure, 3 patients experienced complications, specifically obstruction of the drainage tube, detachment of the drainage tube, and intractable pain at the puncture site. The occurrence of drainage tube obstruction may be linked to the choice of tube size because this patient underwent PTCD under emergency bedside ultrasound guidance, and a 6 Fr drainage tube was selected. This observation resonates with other cases of PTCD tube obstruction encountered in clinical practice. However, given the limited sample size, this hypothesis could not be definitively confirmed at this stage. Detachment of the drainage tube in the patient was attributed to our failure to conduct weekly dressing changes and resecure the PTCD tube, compounded by the patient limited mobility due to advanced age. In patients who experience pain at the puncture site, discomfort stems from the puncture site itself. Specifically, the target bile duct for this patient was segment 8, positioning the puncture point between the ribs at the right anterior axillary line, an area where the intercostal nerves are located. Conversely, patients undergoing puncture of the left liver reported less pain. Our treatment approach involved local anesthesia, prompt PTCSL, and early postoperative removal of the drainage tube. Regrettably, despite complete removal of the stones, the patient bile duct stenosis remained unresolved due to the short duration of biliary dilatation. Furthermore, no established standard currently exists for selecting the puncture site in percutaneous lithotripsy. Various studies have proposed different puncture site locations, necessitating further investigations.

In the 2nd-step, 3 cases of PTCSL-related complications emerged: intraoperative hypothermia, bleeding from dilated sinus tracts during surgery, and postoperative watery diarrhea. Notably, both intraoperative hypothermia and postoperative diarrhea were associated with excessive water infusion during surgery. The occurrence of intraoperative hypothermia in 1 patient was also attributed to inadequate waterproofing measures in the surgical area during the procedure because large area of the patient skin was submerged in the irrigation fluid without our awareness. Consequently, due to the intraoperative hypothermia, the patient was unable to undergo complete stone removal in a single surgery. Subsequently, the patient declined further stone removal procedures, leading to incomplete stone extraction. Bleeding encountered during sinus tract dilation may originate from sources including the intercostal artery, hepatic artery, or portal vein.^[16]^ Treatment options include compression hemostasis, local pharmacological hemostasis, transarterial embolization, and open surgical hemostasis.^[17]^ However, in this patient, bleeding was successfully controlled through manual compression during the procedure, the use of an expanded sheath, and postoperative drainage tube compression. Further analysis indicated that successful hemostasis achieved solely through compression may be attributed to the ruptured vessel being a peripheral branch of the hepatic vasculature because we avoided the main intrahepatic vascular trunk during the initial PTCD puncture. Nevertheless, the correlation between puncture site selection and perioperative bleeding warrants further investigation with a larger sample size.

Beyond the technical success, our study underscores the critical impact of patient psychology on clinical decision making. Following the 1st-step PTCD, a significant improvement in patients systemic symptoms was typically achieved. Particularly when common tube-related issues such as fluid loss or reduced appetite did not manifest, a tendency to delay the definitive PTCSL procedure was observed in some patients. This deferral, which contributed to the prolonged interval between the 2 steps, was primarily driven by a fear of general anesthesia, apprehension about the risks of surgery, and a sense of satisfaction with their improved, albeit temporary, symptomatic relief. We conceptualize this phenomenon as “treatment inertia,” which presents a significant barrier to adhering to the planned treatment timeline. Therefore, future management protocols must extend beyond surgical technique to incorporate enhanced patient counseling, shared decision making, and psychological support, ensuring that patients can access the full benefits of definitive stone clearance without undue delay.

The present investigation had some limitations, the first of which were its retrospective design and relatively small sample size, which may have introduced cohort heterogeneity. Second, due to the lack of research data addressing the application of this technology in elderly patients and the high risk associated with their treatment, we adopted a relatively conservative approach in clinical practice. Third, few patients underwent long-term follow-up, making it exceedingly difficult to accurately evaluate the effects of long-term follow-up on patients. Finally, we found that in actual clinical practice, due to issues related to the treatment attitude of elderly patients, we often could not fully adhere to the initially formulated plan. This was a factor that we had not previously considered. However, we are gradually improving the application mode of this technology in elderly and high-risk patients while ensuring safety of the entire treatment process.

In conclusion, while managing elderly patients with complex cholelithiasis remains challenging, our findings offer a potential pathway. Despite the limitations inherent in a small, retrospective, single-center design, our study suggests that 2-step PTCSL is a promising option, demonstrating safety, and feasibility. However, these preliminary results mandate confirmation in future prospective, comparative studies to robustly establish its efficacy and role in clinical practice.

## Acknowledgments

The authors acknowledge Ms. Juan Chen from the Party Committee Office of The First People’s Hospital of Neijiang for her assistance in language editing.

## Author contributions

**Conceptualization:** Pan Liu, Ji-Lin Zhang, Xin Xiang.

**Data curation:** Ji-Lin Zhang, Xin Xiang.

**Formal analysis:** Sheng Yu, Shun-Hai Liu.

**Funding acquisition:** Pan Liu.

**Resources:** Sheng Yu.

**Software:** Shun-Hai Liu.

**Validation:** Shun-Hai Liu.

**Writing – original draft:** Pan Liu, Ji-Lin Zhang.

**Writing – review & editing:** Pan Liu, Shun-Hai Liu, Xin Xiang.
